# Angiogenin in Parkinson Disease Models: Role of Akt Phosphorylation and Evaluation of AAV-Mediated Angiogenin Expression in MPTP Treated Mice

**DOI:** 10.1371/journal.pone.0056092

**Published:** 2013-02-07

**Authors:** Trent U. Steidinger, Sunny R. Slone, Huiping Ding, David G. Standaert, Talene A. Yacoubian

**Affiliations:** 1 Department of Neurobiology, University of Alabama at Birmingham, Birmingham, Alabama, United States of America; 2 Center for Neurodegeneration and Experimental Therapeutics, Department of Neurology, University of Alabama at Birmingham, Birmingham, Alabama, United States of America; Emory University, United States of America

## Abstract

The angiogenic factor, angiogenin, has been recently linked to both Amyotrophic Lateral Sclerosis (ALS) and Parkinson Disease (PD). We have recently shown that endogenous angiogenin levels are dramatically reduced in an alpha-synuclein mouse model of PD and that exogenous angiogenin protects against cell loss in neurotoxin-based cellular models of PD. Here, we extend our studies to examine whether activation of the prosurvival Akt pathway is required for angiogenin's neuroprotective effects against 1-methyl-4-phenylpyridinium (MPP+), as observed in ALS models, and to test the effect of virally-mediated overexpression of angiogenin in an *in vivo* PD model. Using a dominant negative Akt construct, we demonstrate that inhibition of the Akt pathway does not reduce the protective effect of angiogenin against MPP+ toxicity in the dopaminergic SH-SY5Y cell line. Furthermore, an ALS-associated mutant of angiogenin, K40I, which fails to induce Akt phosphorylation, was similar to wildtype angiogenin in protection against MPP+. These results confirm previous work showing neuroprotective effects of angiogenin against MPP+, and indicate that Akt is not required for this protective effect. We also investigated whether adeno-associated viral serotype 2 (AAV2)-mediated overexpression of angiogenin protects against dopaminergic neuron loss in the 1-methyl-4-phenyl-1,2,3,6-tetrahydropyridine (MPTP) mouse model. We found that angiogenin overexpression using this approach does not reduce the MPTP-induced degeneration of dopaminergic cells in the substantia nigra, nor limit the depletion of dopamine and its metabolites in the striatum. Together, these findings extend the evidence for protective effects of angiogenin *in vitro*, but also suggest that further study of *in vivo* models is required to translate these effects into meaningful therapies.

## Introduction

The potent angiogenic factor, angiogenin, has recently been associated with neurodegenerative diseases, such as Amyotrophic Lateral Sclerosis (ALS) and Parkinson Disease (PD) [1], [2]. Angiogenin was first linked to ALS through genetic studies that revealed the association of certain angiogenin mutations with both sporadic and familial forms of ALS [1], [2], [3], [4], [5], [6], [7], [8], [9]. Wildtype angiogenin has been shown to reduce motoneuron cell death in response to hypoxia, serum deprivation, ER stress, and excitotoxicity, while mutant forms of angiogenin associated with ALS fail to reduce toxicity in these models [10], [11], [12]. Angiogenin treatment delays motor dysfunction and motor neuron loss, and prolongs survival in the superoxide dismutase 1 (SOD1) mouse model of ALS [10]. More recently, angiogenin has been linked to PD. Our lab previously demonstrated a robust down-regulation of angiogenin expression in transgenic mice overexpressing human alpha-synuclein, a mouse model of PD [13], [14]. We also demonstrated that exogenous angiogenin reduced toxicity by rotenone and 1-methyl-4-phenylpyridine (MPP+) in neuroblastoma cell lines [14]. Just recently two genetic screens showed several angiogenin variants to be associated with PD [2], [15].

How angiogenin may promote cell survival in neurodegeneration is not well understood. Angiogenin has been identified to induce several signaling pathways associated with survival and cellular maintenance [10], [16], [17], [18]. In endothelial and smooth muscle cells, angiogenin is linked to the induction of several pathways including stress-associated protein kinase/c-Jun N-terminal kinase (SAPK/JNK), phospholipase C (PLC), extracellular signal-related kinase 1/2 (ERK1/2), and Akt [19], [20], [21], [22]. Angiogenin is also known to localize to the nucleus where it induces rRNA translation and pro-survival protein expression [17], [23].

Activation of the PI3K/Akt pathway has been linked to angiogenin's neuroprotective effects in ALS models. In motoneurons and in the SOD1 ALS mouse model, angiogenin induced phosphorylation of Akt, whose activation was required for angiogenin's neuroprotective effect. In contrast, the ALS-associated mutant K40I failed to induce Akt phosphorylation and failed to be protective in motoneurons [10]. We previously demonstrated angiogenin to induce Akt phosphorylation in the SH-SY5Y dopaminergic cell line [14]. Here we investigate whether activation of the PI3K-Akt pathway is required for angiogenin's protective effect against MPP+ toxicity in SH-SY5Y cells. We also extend our previous studies to test whether virally-mediated overexpression of angiogenin reduces cell loss in the MPTP mouse model, a commonly used neurotoxin model of PD that selectively induces dopaminergic neuronal loss in the substantia nigra and dopamine depletion in the striatum [24].

## Methods

### Cell culture

SH-SY5Y neuroblastoma cells were a gift from J. Zhang (Birmingham, AL), originally obtained from the American Type Culture Collection (ATCC, Manassas, VA) and were grown in Dulbecco's modified Eagle's medium (DMEM) supplemented with 10% fetal bovine serum (FBS). Cells were cultured at 37°C with 5% CO2 in a humidified incubator.

### Recombinant angiogenin

Wildtype human angiogenin was purchased from R & D Systems (Minneapolis, MN) for the dominant negative Akt experiment. Recombinant human wildtype angiogenin and K40I mutant angiogenin were prepared by the UAB Center for AIDS Research Virology Core Molecular Biology Lab (P30AI027767). This mutant and wildtype angiogenin proteins were used for the experiment testing the effect of the K40I mutant on MPP+ toxicity.

### Transfection

Dominant negative (DN)-Akt (K179M) in the pCMV5 plasmid, along with the empty pCMV5 vector, was obtained from Addgene (Cambridge, MA). SH-SY5Y cells were transfected with the DN-Akt or the empty pCMV5 plasmid using the Amaxa nucleofector (Lonza, Walkersville, MD) under the D-017 protocol. Two million cells were used for each transfection with 5 µg of plasmid.

### Western blot analysis

SH-SY5Y cells were sonicated in lysis buffer (150 nM NaCl, 10 mm Tris-HCl (pH 7.4), 1 mM EGTA, 1 mM EDTA, 0.5%NP-40, protease inhibitor cocktail (Roche Diagnostics, Indianapolis, IN)) and centrifuged at 16000 g for 10 min at 4°C. Protein concentrations of supernatants were determined using the bicinchoninic assay (Pierce, Rockford, IL). Each sample was boiled for 5 min in a 4× DTT sample loading buffer (8% SDS, 0.25M Tris-HCl, 200 mM DTT, 30% glycerol, and Bromophenol Blue), resolved on 12% SDS-polyacrylamide gels, and transferred electrophoretically to 0.45-µm nitrocellulose membranes at 100 V for 1 hour. Following transfer, membranes were incubated for 30 min in 5% non-fat dry milk in TBST (25 mM Tris-HCl pH 7.6, 137 mM NaCl, 0.1% Tween 20) and then incubated overnight at 4°C in mouse monoclonal antibody against HA (1∶1000 Covance, Emeryville, CA), rabbit polyclonal antibody against Akt phosphorylated at serine 473 (1∶1000 Cell Signaling, Danvers, MA), rabbit polyclonal antibody against cleaved caspase 3 antibody (1∶1000 Cell Signaling, Danvers, MA), or mouse monoclonal antibody against α-actin (1∶10000; Sigma, St. Louis, MO). After three washes in TBST, blots were incubated for two hours with HRP-conjugated goat anti-rabbit or anti-mouse secondary antibodies (1∶2000; Jackson ImmunoResearch, West Grove, PA) and then washed in TBST six times for ten minutes each. Enhanced chemiluminescence (Pierce, Rockford, IL) was used to detect protein bands. Quantification of protein bands was performed using densitometry, and each band was normalized to the average of all bands.

### Immunocytochemistry in SH-SY5Y cells

SH-SY5Y cells were washed three times in TBS and then fixed in 4% paraformaldehyde. After a rinse in TBS, cells were permeabilized in 0.5% Triton X-100 for 20 minutes and then blocked in 1.5% normal donkey serum for one hour. After incubation in goat anti-angiogenin primary antibody (R & D Systems) in blocking buffer, cells were rinsed three times in TBS and incubated in CY3-conjugated donkey anti-goat secondary antibody for one hour. After three washes in TBS, cells were mounted in Vectashield (Vector Laboratories, Burlingame, CA). 10 random fields per well were imaged by confocal microscopy, and intensity was quantified using Image J [25].

### Viral vector construct

The construction of the rAAV2-CBA-IRES-EGFP-WPRE (CIGW) plasmid has previously been described [26]. The DNA sequence corresponding wildtype human angiogenin with a V5-His epitope tag at the C-terminal end was amplified by PCR using the following primers: 5′-GAATTCGAGCTCGGAAGAAGCGGGTGAGAAAC-3′ (sense) and 5′-GAATTCGTCGACTCAATGGTGATGGTGATGATG-3′ (antisense). The amplified sequence was then digested and ligated into the multiple cloning site (SalI and SacI) of the rAAV2 expression vector. The integrity of the AAV-Ang clone was verified by sequencing. Viral vectors were packaged at the Vector Core of University of North Carolina at Chapel Hill.

### Virus injection

Mice were used in accordance with the guidelines of the National Institute of Health (NIH) and University of Alabama at Birmingham (UAB) Institutional Animal Care and Use Committee (IACUC). Animal work performed in this study was approved by UAB's IACUC (animal protocol #120108979). Eight-week-old male C57BL/6 mice were anesthetized with 3% isoflurane and then placed into a stereotactic frame (Kopf, Tujunga, CA). Anesthesia was maintained using 1.5–2% isoflurane in oxygen through a nose tip built into the stereotactic frame. The tip of 5.0-µl syringe (Hamilton Company, Reno, Nevada) was inserted using the following stereotactic coordinates: anterior-posterior, −3.2 mm from bregma; medio-lateral, −1.2 mm from mid-line; and dorso-ventral, −4.6 mm from the dura. Injections were performed using 2 µl of AAV2-Angiogenin (7.5×10^11^ viral genome/ml) or AAV2-Green Fluorescent Protein (GFP) (9×10^9^ viral genome/ml) at a rate of 0.25 µl/min. The needle was removed two minutes after injection.

### MPTP

MPTP handling and safety measures were in accordance with the UAB's IACUC guidelines. Four weeks following AAV2 injection, mice were subjected to a subacute MPTP regimen. MPTP was administered intraperitoneally every day at a dose of 30 mg/kg of body weight for five days. Mice were anesthetized with ketamine and xylazine and then sacrificed three weeks following the last injection. As control, some AAV2-Ang and AAV2-GFP mice were injected with saline intraperitoneally. Two rounds of injections were performed. In the first round, all animals were used for HPLC analysis and a subset for stereology. In the second round, all animals were used for stereology.

### Tissue preparation for HPLC

Animals were sacrificed three weeks following the last MPTP injection. Striatum was dissected and immediately placed on dry ice for high pressure liquid chromatography (HPLC) analysis. These samples were shipped on dry ice to the Neurochemistry Core Lab at Vanderbilt University Medical Center, Nashville, TN for HPLC analysis of dopamine (DA), 3,4-dihydroxyphenylacetic acid (DOPAC), and homovanillic acid (HVA) in the striatum. A total of 6–10 animals were analyzed for striatal DA metabolites per group.

### Immunohistochemistry

Posterior portions of the brains including the midbrain were rinsed in PBS, fixed by immersion in 4% paraformaldehyde for 3 days, and then incubated in 30% sucrose for 2 days. Using a microtome brains were sliced into 40 µm sections and stored in 50% glycerol in PBS. Free-floating substantia nigra slices were treated with 3% hydrogen peroxide to quench endogenous peroxidases. Sections were then blocked in 10% normal goat serum (NGS) for 30 min, and incubated in primary rabbit polyclonal anti-tyrosine hydroxylase (TH) antibody (1∶1000 Pelfreeze Biologicals, Rogers, AR) in 2% NGS overnight at 4°C. Sections were rinsed in PBS, blocked again in 10% NGS, and incubated in goat anti-rabbit secondary antibody conjugated with horseradish peroxidase (1∶500 Jackson Immunoresearch, West Grove, PA) in 2% NGS for an hour at room temperature. Chromogen staining was displayed with the use of diaminobenzidine substrate kit (Vector Laboratories, Burlingame, CA). To identify localization and expression of virally-injected cells, free floating sections were incubated with primary rabbit anti-green fluorescent protein (1∶1000 Abcam, Cambridge, MA) or rabbit anti-V5 antibodies (1∶1000 Sigma, St. Louis, MO) overnight followed by Alexa-488 conjugated goat anti-rabbit (1∶500 Invitrogen, Carlsbad, CA) or CY3 conjugated goat anti-rabbit antibodies (1∶500 Jackson ImmunoResearch, West Grove, PA)

### Stereological analysis

For each animal, every fourth section through the substantia nigra was analyzed. Using the Olympus BX51 brightfield microscope, substantia nigra regions were scanned and contours traced at low power objective. TH-positive cells were counted with an optical fractionator from the Stereoinvestigator 7.0 software from MBF Biosciences (Microbrightfield Inc, Williston, VT). The number of TH-positive neurons was determined in each counting frame using the optical dissector method, and section thickness was determined to correct for variation between tissue thicknesses. A total of 4–17 animals were analyzed for stereology per group (n = 7 for saline GFP group, n = 4 for saline Ang group, n = 17 for MPTP GFP group, and n = 16 for MPTP Ang group).

### Statistical analysis

All statistical analysis of experiments was performed with GraphPad Prism5 (GraphPad Inc., LaJolla, CA). LDH assay, Western blot analysis, HPLC data, and stereology data was analyzed using one-way ANOVA with a post-hoc Tukey's test. Angiogenin staining intensity data was analyzed using Student's t-test.

## Results

### Akt inhibition does not inhibit angiogenin's protective effects against MPP+

**Figure 1:**
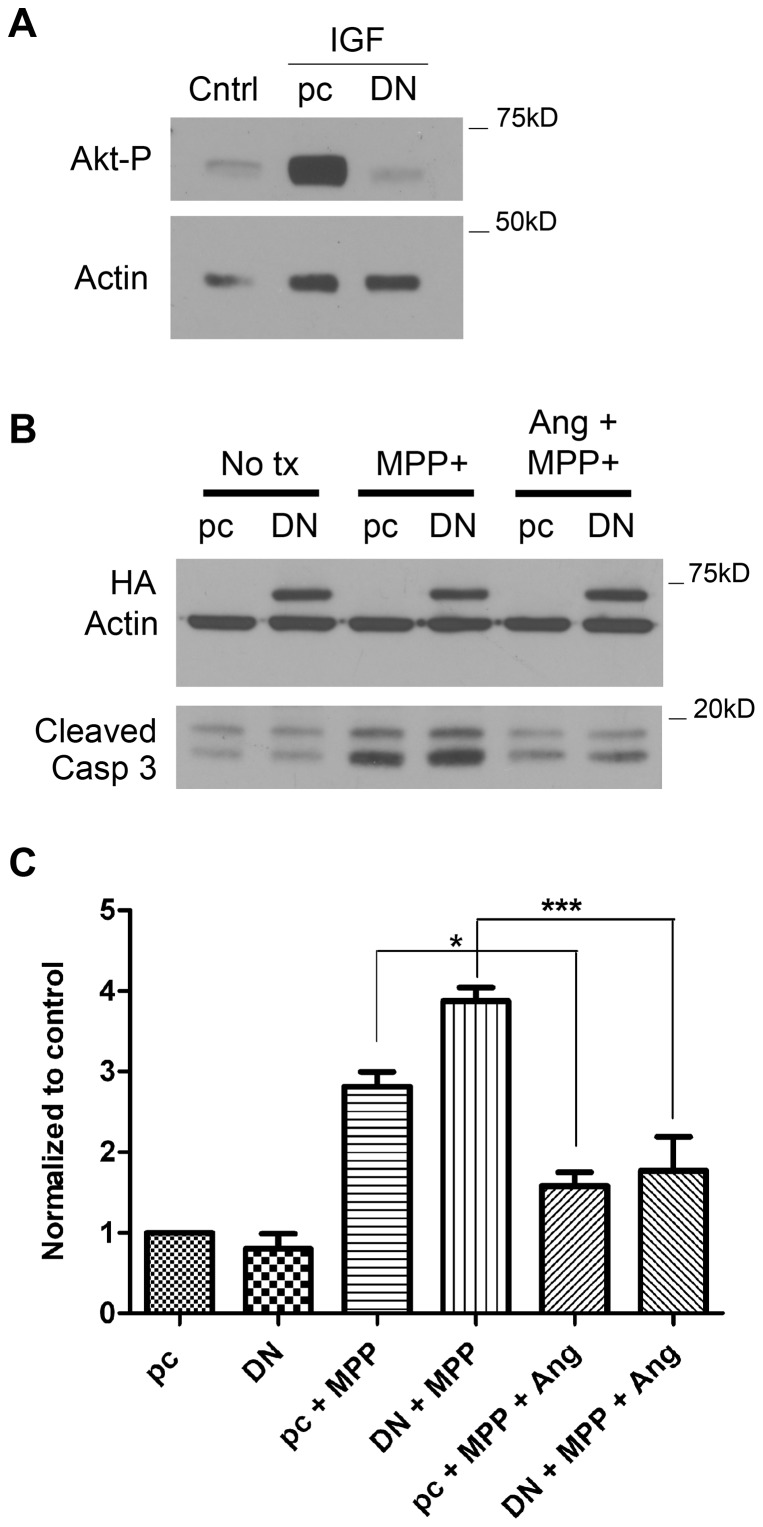
Dominant negative Akt does not inhibit angiogenin's protective effect against MPP+. A. Western blot demonstrating reduced Akt phosphorylation in SH-SY5Y cells transfected with the K179M DN-Akt construct. Twenty-four hours following transfection of empty pCMV5 vector (pC) or DN-Akt vector (DN), SH-SY5Y cells were treated with Insulin Growth Factor (50 ng/mL). Thirty seconds after Insulin Growth Factor treatment, cell lysates were collected for Western blotting. Actin was used as a loading control. B. Representative Western blot demonstrating reduced caspase-3 cleavage following angiogenin treatment in SH-SY5Y cells transfected with either empty vector or HA-tagged DN-Akt using Amaxa nucleoporation. Twenty-four hours after transfection, cells were pretreated with angiogenin (100 nM). MPP+ (0.75 mM) was then applied with fresh angiogenin for an additional 24 hours prior to collection. HA-tagged DN-Akt expression was comparable among all DN-Akt-transfected conditions. Actin was used as loading control. C. Densitometric quantification of caspase-3 cleavage bands normalized to actin. Results reflect three independent experiments. *p<0.05, ***p<0.001 (One-way ANOVA with Tukey's post-hoc test). Error bars reflect SEM.

We have previously shown that exogenous, wildtype angiogenin reduces cell death in response to the neurotoxin MPP+ in neuroblastoma cells [14]. We have also observed that angiogenin treatment induces Akt phosphorylation in SH-SY5Y cells [14]. To investigate if angiogenin's protective effect against MPP+ is dependent on Akt activation, we tested whether inhibition of this pathway would impede the protective response. We used the kinase defective mutant of Akt (K179M), which acts as a dominant negative inhibitor to block Akt signaling [27], [28], [29], [30]. We first show that this dominant negative Akt (DN-Akt) eliminates Akt phosphorylation in response to Insulin Growth Factor in cells transfected with DN-Akt compared to empty vector (Fig. 1a). We next tested whether angiogenin still reduced cell loss in response to MPP+ in SH-SY5Y cells transfected with DN-Akt. 24 hours after transfection with DN-Akt or empty vector, cells were first pretreated with or without angiogenin (100 nM) for 12 hours and then MPP+ (0.75 mM) was added to both control and angiogenin-treated cells for another 24 hour incubation. As an additional control, some cells transfected with either empty vector or DN-Akt were not treated with either angiogenin or MPP+. Cell death was then assessed by caspase-3 cleavage by Western blot. Our results indicate that angiogenin is still protective against MPP+ in the presence of DN-Akt (Fig. 1b,c), suggesting that Akt activation is not critical for angiogenin's effect in the SH-SY5Y dopaminergic cell line.

### K40I mutant fails to induce Akt phosphorylation but remains neuroprotective

**Figure 2:**
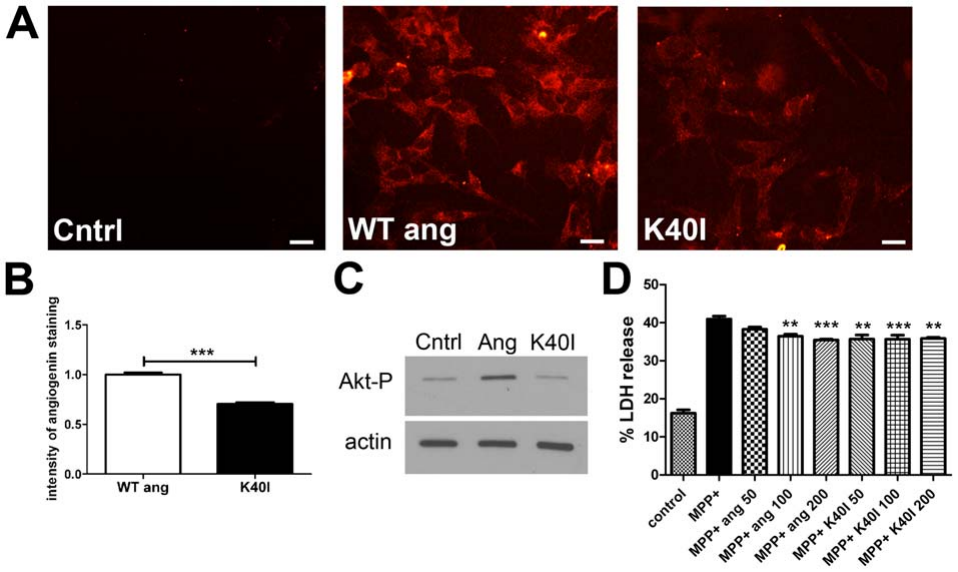
K40I fails to activate Akt phosphorylation but protects against MPP+. A. Immunostaining of SH-SY5Y cells treated with exogenous angiogenin (100 nM) or K40I (100 nM) for 12 hours. Control is immunostaining of SH-SY5Y cells not treated with recombinant angiogenin and reflects low endogenous angiogenin levels in SH-SY5Y cells. Both wildtype and K40I recombinant angiogenin are taken up by SH-SY5Y cells. As the intensity in staining is stronger for wildtype angiogenin compared to K40I, either less K40I is taken up compared to wildtype or the antibody used better recognizes the wildtype form. Scale bar = 25 µm. B. Quantification of angiogenin immunostaining of SH-SY5Y cells treated with exogenous wildtype or K40I angiogenin. 10 random fields per well were imaged by confocal microscopy, and intensity was quantified using Image J. Average of 30 cells was analyzed per field, and two separate immunostaining runs were quantified. ***p<0.001 (students' t-test). C. Western blot demonstrating increased Akt phosphorylation after 30 sec treatment with recombinant angiogenin (100 nM) but not with the K40I angiogenin mutant (100 nM) in SH-SY5Y cells. Actin was used as the loading control. D. LDH assay of SH-SY5Y cells treated with MPP+ with and without wildtype or K40I angiogenin. Cells were pretreated with wildtype or K40I angiogenin (50–200 nM) for 12 hrs and then co-incubated with MPP+ (0.75 µM) and fresh angiogenin or K40I for another twenty-four hours. Angiogenin demonstrated a protective effect that was not significantly different from K40I. n = 4–6, **p<0.01, ***p<0.001 compared to MPP+ treatment (One-way ANOVA with Tukey's post-hoc test). There is no significant difference between wildtype and K40I-treated conditions. Error bars reflect SEM.

The ALS-associated mutant K40I lacks neuroprotective effects compared to wildtype angiogenin in ALS models including hypoxia and serum deprivation [10], [11], [31]. While the mechanism whereby K40I is ineffective compared to wild-type angiogenin is not fully understood, it has been postulated to be a result of its inability to phosphorylate Akt [10], [11]. Since Akt activation is not required for angiogenin's protective effect based on our DN-Akt experiment, we investigated whether the K40I mutant is protective against MPP+. We first tested whether K40I angiogenin can be taken up by SY-SY5Y cells. SH-SY5Y cells were treated with wildtype or K40I angiogenin for 12 hours and then stained using an antibody against angiogenin. Endogenous levels of angiogenin are low in SH-SY5Y cell, as demonstrated in control wells that were not treated with recombinant protein (Fig. 2a panel 1). K40I mutant is able to be taken up by SH-SY5Y cells (Fig. 2a), but the intensity of staining for angiogenin is less for the K40I mutant than wildtype angiogenin (Fig. 2a,b). This suggests that either K40I is not taken up as efficiently as wildtype angiogenin in these cells, or alternatively the angiogenin antibody may detect wildtype angiogenin better than the mutant.

We then tested whether K40I mutant could induce Akt phosphorylation in SH-SY5Y cells. Wildtype angiogenin (100 nM) and mutant K40I angiogenin (100 nM) were applied to SH-SY5Y cells for 30 seconds. Cells were then collected and analyzed for phosphorylated Akt at serine 437 by western blot. As expected, the mutant K40I failed to induce Akt phosphorylation compared to wildtype (Fig. 2c). We then investigated whether mutant K40I could reduce MPP+ toxicity in SH-SY5Y cells. SH-SY5Y cells were pretreated with either wildtype angiogenin or mutant K40I (50–200 nM) for 12 hours and then co-treated with MPP+ and wildtype or K40I angiogenin for another 24 hours. Cell death was determined by LDH release into the media. Wildtype angiogenin showed mild protection against MPP+ in a dose-dependent manner. Cells treated with mutant K40I showed no significant difference in MPP+-induced cell death compared to cells treated with wildtype angiogenin (Fig. 2d).

### Virally-mediated overexpression of angiogenin does not reduce dopaminergic loss in the MPTP mouse model

**Figure 3:**
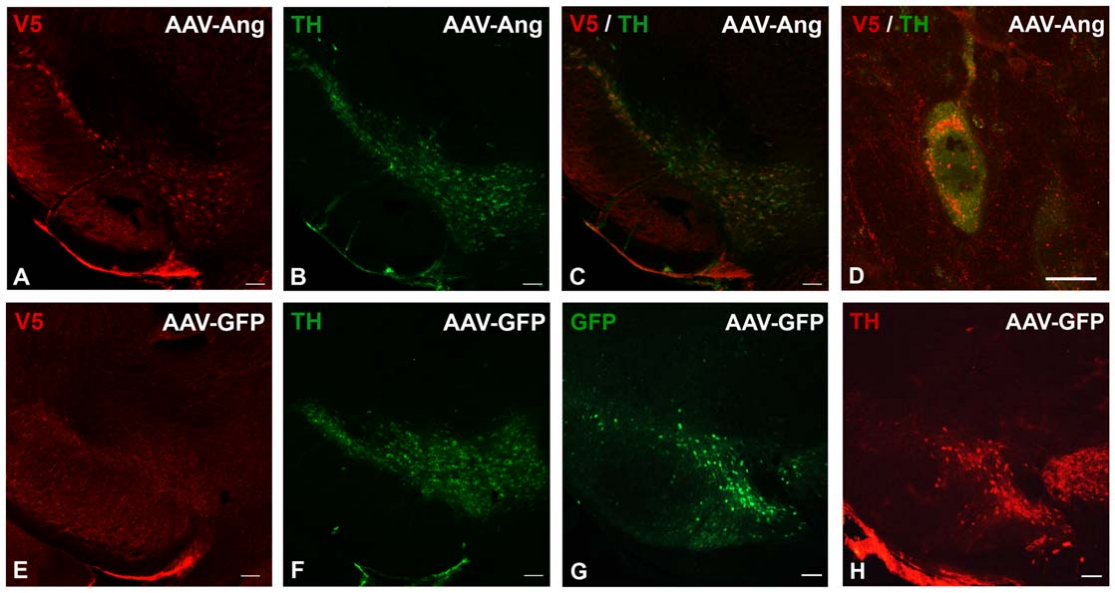
Angiogenin is expressed in TH-positive neurons in the substantia nigra after viral injection. A. An AAV2-Ang injected mouse was sacrificed eight weeks after injection and stained with a primary antibody against the V5 tag fused to angiogenin and a CY3-conjugated secondary antibody, demonstrating overexpression of angiogenin in the substantia nigra. Scale bar = 100 µm. B. Same section of tissue stained with a primary antibody against tyrosine hydroxylase (TH) and an Alexa488-conjugated secondary antibody to identify dopaminergic nigral neurons. Scale bar = 100 µm. C. A merge of V5-angiogenin staining and TH staining demonstrating colocalization in dopaminergic cells in the substantia nigra. Scale bar = 100 µm. D. Higher magnification image demonstrating V5-angiogenin localized perinuclearly and accumulated in vesicular-like puncta within the cell. Scale bar = 10 µm. E. Control immunostaining of the substantia nigra from an AAV2-GFP injected mouse. No V5-angiogenin staining was observed. Scale bar = 100 µm. F. Same section stained for TH to identify dopaminergic neurons in the substantia nigra of an AAV2-GFP-injected mouse. Scale bar = 100 µm. G. GFP immunostaining of the substantia nigra from another section from the same AAV-GFP injected mouse, demonstrating GFP expression in the substantia nigra. H. Same section stained for TH to identify dopaminergic neurons in the substantia nigra of an AAV-GFP injected mouse. Scale bar = 100 µm.

We next investigated whether virally-mediated overexpression of angiogenin in the substantia nigra could reduce MPTP toxicity in mouse. We cloned human wildtype angiogenin tagged at the C-terminal end with a V5-His epitope tag into an adeno-associated virus 2 (AAV2) construct containing green fluorescent protein (GFP) [26]. GFP is still expressed by this virus due to presence of the preserved internal ribosomal entry site. Eight-week-old male mice were stereotactically injected with either AAV2-GFP or AAV2-angiogenin (AAV2-Ang) virus into the right substantia nigra and then sacrificed for immunostaining with an antibody against V5 eight weeks after injection. We identified modest angiogenin expression co-localized with tyrosine hydroxylase (TH) in the substantia nigra at eight weeks after injection (Fig. 3a–c). Intracellularly, overexpressed angiogenin demonstrated localization in a perinuclear pattern within vesicular-like puncta (Fig. 3d). GFP staining but not V5 staining was not observed in TH-positive nigral neurons that were infected with AAV2-GFP (Fig. 3e–h).

**Figure 4:**
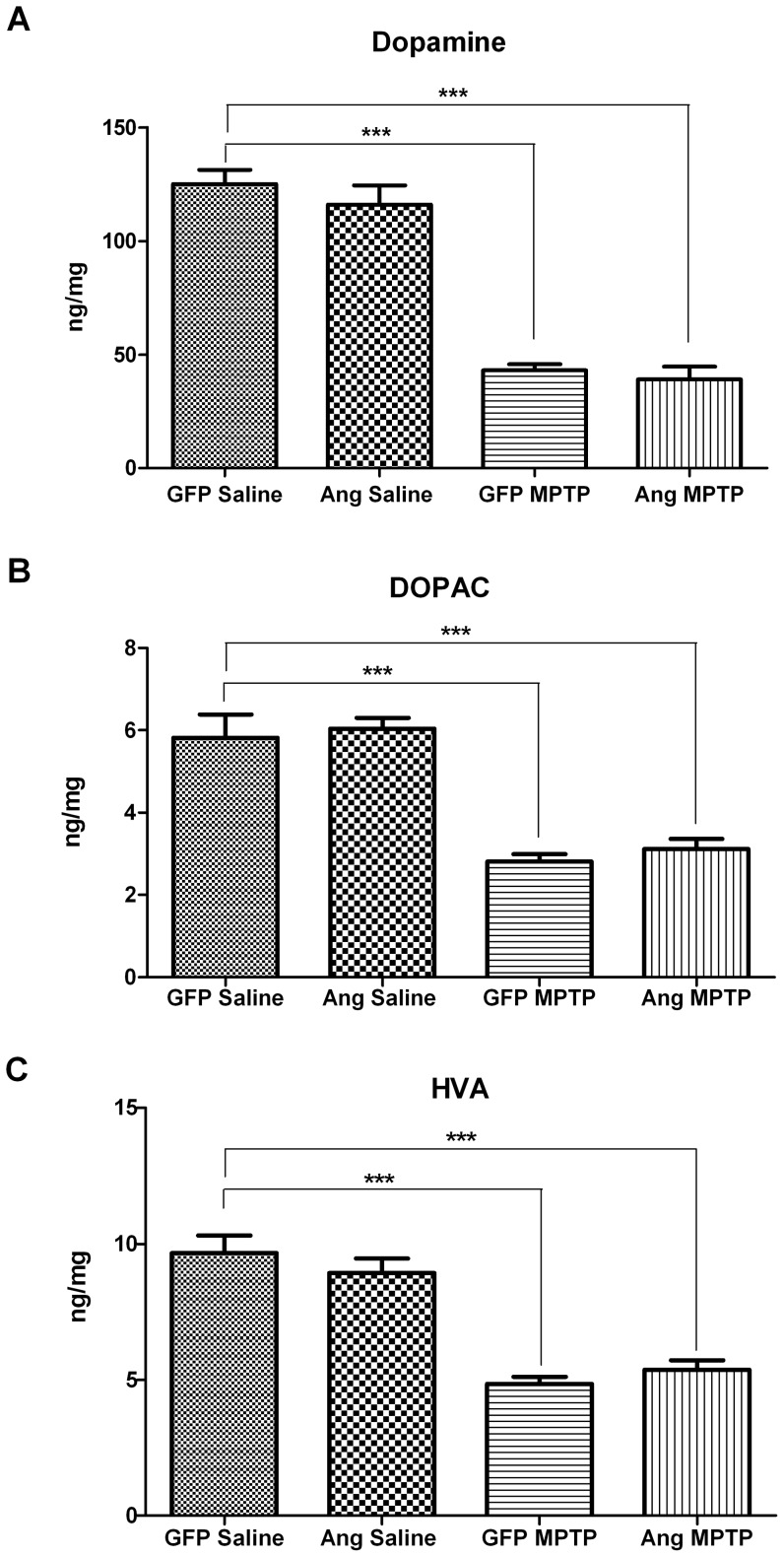
Angiogenin does not prevent MPTP-induced depletion of dopamine and its metabolites in the striatum. Three weeks after MPTP or saline injections, AAV2-GFP and AAV2-Ang-injected mice were sacrificed and striatal tissue immediately removed and frozen for HPLC. DA and its metabolites, DOPAC and HVA, were analyzed by HPLC. GFP-Saline n = 10, Ang-Saline n = 10, GFP-MPTP n = 9, Ang-MPTP n = 6. Error bars reflect SEM. A. Striatal dopamine levels as determined by HPLC. AAV2-Ang mice treated with MPTP showed a comparable loss in striatal DA. ***p<0.001 (One-way ANOVA with Tukey's post-hoc test). B. Striatal DOPAC levels as determined by HPLC. A similar reduction in DOPAC was seen in MPTP-treated AAV2-Ang injected mice as compared to AAV2-Ang MPTP. ***p<0.001 (One-way ANOVA with Tukey's post-hoc test). C. Striatal HVA levels as determined by HPLC. AAV2-Ang-injected mice treated with MPTP had a similar decrease in HVA as compared to AAV2-GFP mice treated with MPTP. ***p<0.001 (One-way ANOVA with Tukey's post-hoc test).

To test whether angiogenin overexpression can reduce MPTP toxicity, eight-week-old mice were injected stereotactically with either AAV2-GFP or AAV2-Ang. Four weeks following AAV2 injection, mice were then given daily injections with either saline or MPTP (30 mg/kg/day) for five days total. 21 days after the last MPTP injection, mice were sacrificed and brains analyzed for dopamine metabolites in the striatum and for dopaminergic neuronal counts in the substantia nigra. The striatal DA levels in the MPTP-treated mice injected with AAV2-GFP were significantly reduced to 34% of DA levels in saline-treated mice injected with AAV2-GFP (p<0.001; Fig. 4a). Similarly, DOPAC and HVA levels in the MPTP-treated mice injected with AAV2-GFP were also reduced to 48% (p<0.001) and 50% (p<0.001), respectively, of levels in the saline-injected controls (Fig. 4b, c). The AAV2-Ang mice treated with saline did not demonstrate any differences in DA, DOPAC, or HVA striatal levels compared to AAV2-GFP mice treated with saline, indicating angiogenin did not affect dopamine metabolite levels at baseline (Fig. 4). MPTP-treated mice injected with AAV2-Ang showed a similar decrease in striatal DA and its metabolites HVA and DOPAC, as compared to MPTP-treated AAV2-GFP mice (Fig. 4).

**Figure 5:**
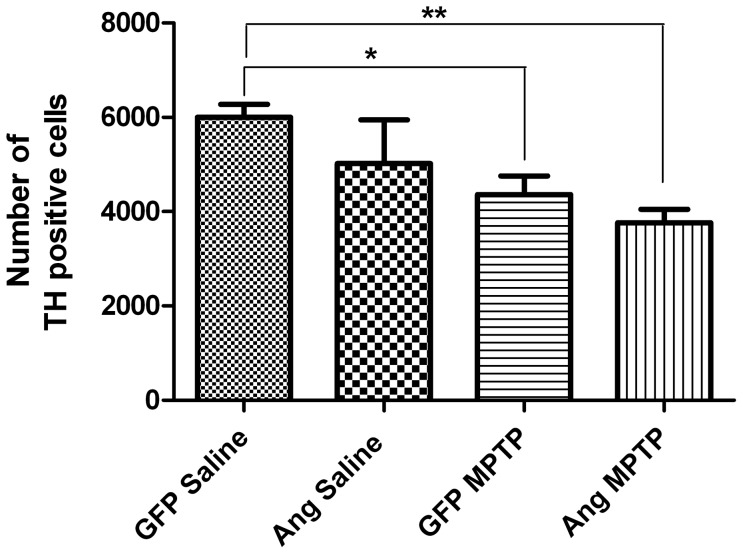
Angiogenin overexpression does not reduce MPTP-induced dopaminergic neuronal loss in the substantia nigra. Four weeks following AAV2 injection, MPTP was administered at 30 mg/kg/day for five days. Mice were sacrificed three weeks following the last MPTP injection and brain sections stained for tyrosine hydroxylase to identify DA neurons in the substantia nigra. Stereology counts of TH-positive nigral neurons in the AAV-injected side are shown for each of the four conditions: GFP-saline (n = 7), Ang-saline (n = 4), GFP-MPTP (n = 17), and Ang-MPTP (n = 16). MPTP induced a 28% reduction in TH-stained neurons in the substantia nigra of GFP-injected mice, and angiogenin did not ameliorate dopaminergic neuron loss induced by MPTP. *p<0.05, **p<0.01 (One-way ANOVA with Tukey's post-hoc test). Error bars reflect SEM.

We next measured nigral dopamine cell counts by stereology in all four mouse groups. TH-positive cell counts were reduced by 27% (p<0.05) in the AAV2-GFP mice following MPTP administration compared to AAV2-GFP treated with saline, as revealed by stereological counts of the AAV2-injected side of the substantia nigra (Fig. 5). TH-positive cell counts in AAV2-Ang mice treated with MPTP were similar to MPTP-treated mice injected with AAV2-GFP. There was no statistically significant difference in TH-positive cell counts in saline-treated mice injected with AAV2-GFP compared to saline-treated mice injected with AAV2-Ang (Fig. 5).

## Discussion

In this study we investigated the role of the Akt survival signaling pathway in angiogenin's neuroprotective response *in vitro*, as well as tested whether virally-mediated overexpression of angiogenin can reduce cell loss in an *in vivo* model of PD. Our results demonstrate that inhibition of Akt activation does not significantly alter angiogenin's neuroprotective effect against MPP+-induced toxicity. Additionally, the K40I mutant that fails to induce Akt phosphorylation was still protective against MPP+ in SH-SY5Y cells. We further demonstrate that the AAV2-driven overexpression of angiogenin failed to reduce MPTP-induced toxicity in mice.

The PI3K/Akt pathway has been implicated for angiogenin's neuroprotective effect in ALS models [10], [11]. Protection by angiogenin against excitotoxicity in motoneurons was eliminated by treatment with the PI3K inhibitor wortmannin. The K40I mutant failed to induce Akt phosphorylation as well as failed to protect against ER stress, serum deprivation, and hypoxia [10], [11]. In addition, Akt phosphorylation was observed in the SOD1 mouse model in response to intraperitoneal injection of angiogenin [10], [11]. In our cellular PD model we also saw increased Akt phosphorylation in response to angiogenin [14]; however, here we demonstrated that Akt is not required for angiogenin's protective effect against MPP+, as DN-Akt failed to eliminate angiogenin's neuroprotective effect. We did test the PI3K inhibitors wortmannin and LY294002 in our cellular system, but they significantly magnified MPP+ toxicity in SH-SY5Y cells, making interpretation of the data difficult (data not shown). We did also observe that DN-Akt increased the toxicity of MPP+ in SH-SY5Y cells (Fig. 1c) but not to the extent observed with PI3K inhibitors. Despite this additional increase in toxicity when MPP+ was combined with DN-Akt, angiogenin was still able to reverse toxicity, suggesting that Akt is not required for protection. We also demonstrate that contrary to wild type angiogenin, K40I did not induce Akt phosphorylation as did wildtype angiogenin, and yet it similarly protected against MPP+ in SH-SY5Y cells. Therefore, our studies suggest that while Akt activation may occur in response to angiogenin treatment in SH-SY5Y cells, Akt activation is not required and other signaling pathways are likely activated in parallel that can also mediate angiogenin's effects.

Several other signaling pathways linked to cell survival have been shown to be induced by angiogenin in other cells, including ERK1/2, NF-κB, phospholipase C, and SAPK/JNK pathways [16], [17], [19], [21], [22]. Angiogenin also can induce expression of anti-apoptotic factors, such as Bcl-2 [17]. K40I is able to localize to the nucleus similar to wild-type angiogenin in the SH-SY5Y cell line [32], suggesting that it could also augment pro-survival gene expression. We did not observe increased Bcl-2 protein levels in response to angiogenin treatment in our cells (data not shown). Angiogenin can also promote cell survival through its ribonuclease activity, which has been directly associated with stress-induced translational repression [33]. However, this ribonuclease activity is unlikely involved in angiogenin's effects against MPP+, as the K40I mutant which lacks ribonuclease activity [34] does protect against MPP+. We suspect that multiple pathways may act in concert to promote cell survival in dopaminergic cell lines such that disruption of one pathway may not be sufficient to block angiogenin's effects against MPP+ *in vitro*.

We additionally investigated if angiogenin was protective in the MPTP mouse model of PD. Using AAV2-Ang to drive overexpression in the substantia nigra of MPTP-treated mice, our results here indicate angiogenin does not inhibit dopaminergic cell death in the substantia nigra nor the depletion of striatal DA following MPTP treatment. We conclude that angiogenin overexpression by AAV2 is not adequate for protection against MPTP, at least in mice. The levels of angiogenin generated by stereotactic AAV2 injection were modest in the substantia nigra, and alternative angiogenin delivery methods may better reach any required therapeutic threshold for angiogenin's potential protective response *in vivo*. Another limitation could be that angiogenin's protective effect in dopaminergic cells may require paracrine signaling that is disrupted by overexpression of angiogenin in the target TH-positive neurons. Recently, Skorupa *et al.* demonstrated that angiogenin promoted motoneuron survival via a paracrine manner involving co-cultured astrocytes [35]. Overexpression of angiogenin directly in TH-positive cells may disrupt paracrine signaling via downregulation of any required receptors and/or signaling molecules. Indeed, in SH-SY5Y cells we found that exogenous delivery of angiogenin induced a protective effect [14], while we found variable protection by angiogenin against MPP+ when SH-SY5Y cells were transfected directly with angiogenin despite good angiogenin expression levels detected by Western blot (data not shown). Finally, although not statistically significant, angiogenin mice injected with saline showed a trend towards lower DA cell counts compared to GFP mice injected with saline. This raises the possibility that angiogenin is not protective against MPTP because it may cause toxicity to nigral neurons at baseline.

In summary we demonstrate that, although Akt is activated by angiogenin, it is not required for angiogenin's neuroprotective effect against MPP+ in SH-SY5Y cells. Our findings suggest that in our SH-SY5Y cell line, wildtype angiogenin and K40I induce another signaling pathway other than Akt that is required for its protective response, or that multiple pathways functioning in parallel act to promote survival such that inhibition of one pathway is not sufficient to block neuroprotection by angiogenin. Further investigation is needed to elucidate the critical components of angiogenin's effect. We further demonstrate that virally-mediated overexpression of angiogenin in the substantia nigra does not reduce toxicity in the MPTP mouse model. Alternative angiogenin delivery methods will need to be explored to determine whether it is possible to translate the *in vitro* protective effects of angiogenin into a useful PD therapy.
